# Loss of CCL28 and CXCL17 Expression and Increase in CCR1 Expression May Be Related to Malignant Transformation of LGBLEL into Lymphoma

**DOI:** 10.3390/cimb46100652

**Published:** 2024-09-29

**Authors:** Rui Liu, Mingshen Ma, Jing Li, Fuxiao Luan, Tingting Ren, Nan Wang, Jianmin Ma

**Affiliations:** 1 Beijing Institute of Ophthalmology, Beijing Tongren Eye Center, Beijing Tongren Hospital, Capital Medical University, Beijing 100730, China; 13476119094@163.com (R.L.); ljlsdlyh@126.com (J.L.); 15668308053@163.com (T.R.); wnnnn1987@163.com (N.W.); 2 Beijing Chaoyang Hospital, Capital Medical University, Beijing 100020, China; mingshen@stanford.edu (M.M.); fxluan@126.com (F.L.)

**Keywords:** lacrimal gland, benign lymphoepithelial lesion, lymphoma, pathogenesis, chemokine signaling pathway

## Abstract

**Abstract:** To investigate the differential expression of the chemokine signaling pathway in lacrimal gland benign lymphoepithelial lesion (LGBLEL) and lacrimal lymphoma, providing insights into the mechanisms underlying malignant transformation and aiding clinical differentiation. Transcriptome analysis was conducted on patients with LGBLEL, lymphoma, and orbital cavernous hemangioma (CH). Three cases of LGBLEL and three cases of lymphoma were randomly selected as control and experimental groups, respectively. A real-time quantitative polymerase chain reaction (RT-qPCR) was used to validate genes associated with the chemokine signaling pathway. Immunohistochemical (IHC) staining and quantitative Western blotting (WB) were performed for precise protein quantification. Transcriptome analysis revealed differential expression of the chemokine signaling pathway between the LGBLEL and lymphoma groups, identifying ten differentially expressed genes: *CCL17*, *VAV2*, *CXCR5*, *NRAS*, *HCK*, *RASGRP2*, *PREX1*, *GNB5*, *ADRBK2*, and *CCL22*. RT-qPCR showed that, compared to the lymphoma group, the LGBLEL group had significantly higher expression of *CCL28*, *CXCL17*, *HCK*, *GNB5*, *NRAS*, and *VAV2* (*p* = 0.001, <0.001, <0.001, <0.001, =0.020, <0.001, respectively) and lower expression of *CCR1* (*p* = 0.002). IHC staining and quantitative analysis confirmed significant differences in protein expression between the groups for CCL28, CCR1, CXCL17, HCK, GNB5, NRAS, and VAV2 (*p* = 0.003, 0.011, 0.001, 0.024, 0.005, 0.019, and 0.031, respectively). While IHC provided localization, WB offered greater precision. WB revealed that, compared to the lymphoma group, the LGBLEL group exhibited significantly higher expression of CCL28, CXCL17, HCK, GNB5, NRAS, and VAV2 (*p* = 0.012, 0.005, 0.009, 0.011, 0.008, and 0.003, respectively) and lower expression of CCR1 (*p* = 0.014). The chemokine signaling pathway plays a role in the malignant transformation of LGBLEL. The decreased expression of CCL28 and CXCL17, coupled with the increased expression of CCR1, may be linked to the progression of LGBLEL into lymphoma.

## 1. Introduction

Lacrimal gland benign lymphoepithelial lesion (LGBLEL), also known as Mikulicz disease, is a chronic inflammatory lesion characterized by the painless enlargement of the lacrimal gland and the swelling of the eyelids [1,2]. With advancements in the understanding of the concept of “immunoglobulin G4-related ophthalmic disease (IgG4-ROD)” in recent years, the Japanese scholar Goto proposed diagnostic criteria for IgG4-ROD in 2014 [3]. IgG4-ROD is a subset of IgG4-related disease (IgG4-RD), with an incidence rate of approximately 4–34% in IgG4-RD and 25% among orbital lymphoproliferative diseases [4,5]. While the lacrimal gland is affected in 84% of IgG4-ROD cases, other orbital structures, such as the trigeminal nerve, extraocular muscles, orbital fat, eyelid, and other tissues, may also be involved [6]. It is suggested that Mikulicz disease, BLEL, and other lymphoplasmacytic infiltrative diseases fall under the umbrella of IgG4-ROD [7].

Research has shown that LGBLEL can progress to lymphoma, with mucosa-associated lymphoid tissue (MALT) lymphoma being the most common type. Other forms, such as diffuse large B-cell lymphoma and follicular lymphoma, have also been reported [8,9]. Additionally, one study found that the incidence of lymphoma in patients with IgG4-ROD is approximately 7%, which is 10 times higher than that in the general population [10]. The mechanisms behind the transformation of LGBLEL into lymphoma are complex but may involve several pathways, including B-cell receptor signaling, NF-κB signaling, FcγR-mediated phagocytosis, FcεRI signaling, pathways in cancer, and the complement system [11,12].

Chemokines and their receptors may play a crucial role in immune and inflammatory responses, as well as in the development and progression of lymphoid tumors. Chemokines regulate B–T cell interactions, and the primary pathological manifestation of LGBLEL and lymphoma is the presence of abundant inflammatory lymphocytes or diffuse infiltration of neoplastic lymphocytes [13,14]. In our previous transcriptome sequencing analysis comparing LGBLEL and cavernous hemangioma (CH), we identified an upregulation of the chemokine signaling pathway in LGBLEL. This upregulation involved 39 differentially expressed genes (DEGs), including RASGRP2, CXCL9, ADRBK2, CCR1, CXCL14, and many others [14]. Based on these findings, we hypothesize that the chemokine signaling pathway may play a crucial role in the development and malignant transformation of LGBLEL. To further explore this, we performed additional transcriptome sequencing of LGBLEL and lymphoma, using CH as a control. According to the characteristics of the disease, lacrimal lymphoma is more common in males, LGBLEL is more likely to occur in females, and CH has a slightly greater tendency to develop in females. Both LGBLEL and CH are more common in middle age, but the age of onset of lacrimal lymphoma is older than that of LGBLEL and CH [15,16]. This article aims to confirm the differential expression of the chemokine signaling pathway in LGBLEL and lymphoma, providing insight into its mechanisms of development and aiding in clinical differentiation.

## 2. Materials and Methods

### 2.1. Subjects

**Figure 1:**
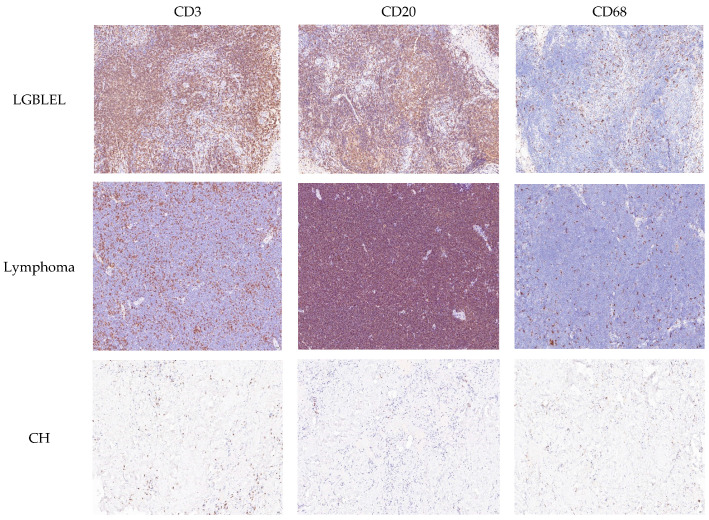
Immunohistochemical staining of CD3, CD20, and CD68 in LGBLEL, lacrimal lymphoma, and CH (×200). CD3 and CD20 exhibited abundant positive expression, while CD68 showed scattered positive expression in the LGBLEL group. CD20 demonstrated strong positive expression, with tumor B cells lacking expression of CD3 and CD68 in the MALT lymphoma group. In the CH group, CD3, CD20, and CD68 were predominantly negative.

The main pathological manifestations of LGBLEL include diffuse infiltration of plasma cells and lymphocytes in the lacrimal gland, glandular atrophy, and fibrous tissue hyperplasia [1,2]. To investigate the immune involvement, we performed immunohistochemical staining on B cells, T cells, and macrophages in LGBLEL, MALT lymphoma, and CH. The results demonstrated higher expression levels of B cells and T cells in both LGBLEL and MALT lymphoma, suggesting that their pathogenesis is closely linked to immune mechanisms (Figure 1). Between January 2011 and January 2012, two experienced pathologists randomly selected 15 cases of LGBLEL, 14 cases of lacrimal lymphoma, and 9 cases of CH, all confirmed by histopathological examination, from Capital Medical University for transcriptome sequencing. Statistically significant differences in age and sex were observed between the LGBLEL and lymphoma groups, while no significant differences were found between the other groups (Table 1 and Table 2). For the verification experiment, 3 cases of LGBLEL and 3 cases of MALT lymphoma diagnosed histopathologically between May and June 2023 were randomly selected as the experimental and the control group, respectively. Consequently, no significant differences in age or sex were found between the LGBLEL and CH groups, but there were notable differences in onset between LGBLEL and lymphoma, as well as between lymphoma and CH.

Inclusion criteria included 1. confirmation by histopathology; 2. no inflammatory or tumor lesions in areas other than the lacrimal gland; and 3. a complete medical history. Exclusion criteria included 1. the presence of inflammatory or tumor lesions in areas other than the lacrimal gland and 2. an incomplete medical history. This study was conducted in accordance with the principles of the Declaration of Helsinki and approved by the Ethics Committee of Beijing Tongren Hospital, Capital Medical University (TRECKY2019-093).

### 2.2. Tissue and Blood Specimen Collection

Pathological tissues from LGBLEL, lymphoma, and CH patients were collected during surgery and transferred to laboratories. Some of the tissue samples were frozen at −80 °C for future use, and the remainder were embedded in paraffin soaked with 10% formalin.

### 2.3. Whole-Transcript Sequencing

We used whole-transcript sequencing to study LGBLEL, lymphoma and CH, with CH as the control group and LGBLEL and Lymphoma as the experimental groups. Total RNA was extracted using a mirVana miRNA Isolation Kit (Ambion, Austin, TX, USA). The quantity and integrity of total RNA samples was assessed using an Agilent 2100 Bioanalyzer, a Qubit 2.0 Fluorometer (Life Technologies, Waltham, MA, USA); cDNA was synthesized, and fragments were enriched by PCR. For sequencing, the Illumina HiSeq (100–2000 bp paired-end) platform and FASTQ software version 0.0.13 (http://hannonlab.cshl.edu/fastx_toolkit/, accessed on 1 September 2018) were used for quality control [17]. Reads were mapped to the human reference genome hg19 (RefSeq annotation) using the STAR aligner (v2.5.0a, Illumina) of the RNA-seq Alignment app (v2.0.1) with default parameters in BaseSpace [18]. The featuresCounts software (v2.0.3) was used to accurately count reads, and the abundance of gene expression was calculated using fragments per kilobase of exon per million fragments mapped (FPKM). The Benjamini–Hochberg method was utilized for multiple tests and corrections to control the false discovery rate (FDR). The DEGs were screened by DESeq and selected based on |log2FC| > 1 and FDR < 0.1. Gene Ontology (GO) enrichment analysis was conducted using the GOseq R software package (v1.12.0). KOBAS software (v2.0) was employed to analyze the statistical enrichment of DEGs in the Kyoto Encyclopedia of Genes and Genomes (KEGG) pathway.

### 2.4. Real-Time Quantitative Polymerase Chain Reaction (RT-qPCR)

RNA was extracted with a TRIzol reagent (Invitrogen). Total RNA was reverse-transcribed into cDNA using the aScript cDNA SuperMix (Quanta Biosciences, Beverly, MA, USA). Gene primers were designed with Primer Express v3.0 Software. PCR reaction conditions were 95 °C/3 min, followed by 40 cycles of 95 °C/30 s, 55 °C/20 s, 72 °C/20 s, each involving DNA denaturation, annealing, and extension [14]. GADPH was used as an endogenous control. Melting curves were monitored to detect nonspecific amplifications. Primer sequence information is shown in Table 3.

### 2.5. Immunohistochemical Staining (IHC)

The tissue sections were placed in xylene for deparaffinization, followed by rehydration through a series of ethanol gradients (100%, 95%, 70%, 50%) and a final rinse with distilled water. After deparaffinization, antigen retrieval was performed in 0.01 M citrate buffer, and endogenous peroxidase activity was quenched by incubating the sections in 3% hydrogen peroxide in methanol for 10 min. To block nonspecific binding, the slides were incubated with 10% normal goat serum in phosphate-buffered saline (PBS) for 1 h at room temperature. Primary antibodies were applied to the slides and incubated overnight at 4 °C, followed by incubation with biotinylated goat antimouse immunoglobulin G (St. Louis, MO, USA) for 1 h at room temperature. Following three washes with PBS, a biotin-labeled secondary antibody was added, and the tissue was incubated at 37 °C/60 min [14]. An appropriate amount of biotin substrate was then added. The slides were colored with DAB solution and subsequently stained with hematoxylin. Finally, the tissue was dehydrated and made transparent with xylene. The immunohistochemical staining results were evaluated by two specialized pathologists. The primary antibodies were as follows: CCL28 (DF7045; Affinity Biosciences, Beijing, China), CXCL17 (18108-1-AP; Proteintech, Wuhan, China), HCK (abs111311; Absin, Shanghai, China), GNB5 (abs141190; Absin), NRAS (abs119341; Absin), VAV2 (abs146517; Absin), CCR1 (abs146517; Absin), and GAPDH (60004-1-1g; Proteintech).

### 2.6. Western Blotting (WB)

Took the lacrimal gland tissue out of the −80 °C freezer, cut a portion of the tissue, and transferred it into a 1.5 mL EP tube, then minced it. Added 250 μL of Western and IP lysis buffer to each tube and lysed the tissue on ice for 45 min. After lysis, centrifuged the tissue at 12,000 rpm for 15 min at 4 °C, extracted the protein supernatant, and quantified it. Loaded all protein samples in 5 × sodium dodecyl sulfate–polyacrylamide gel electrophoresis (SDS–PAGE) loading buffer (P1040, Solarbio, Beijing, China), heated them in a 100 °C water bath for 10 min, and prepared them for SDS–PAGE and PVDF membrane transfer [14]. Soaked the PVDF membrane in TBST containing 5% skim milk (blocking solution) and blocked it on a shaker at room temperature for 2 h. Diluted the corresponding primary antibody with the blocking solution and incubated the PVDF membrane in the primary antibody incubation solution at 4 °C overnight. Washed the PVDF membrane thoroughly with TBST 3–4 times, 15 min per wash. Diluted the HRP-labeled secondary antibody with the blocking solution and incubated the PVDF membrane in the secondary antibody incubation solution at 37 °C on a shaker for 2 h. Washed the PVDF membrane thoroughly with TBST 3–4 times, 15 min per wash. Mixed the enhanced ECL reagent and stable peroxidase solution in a 1:1 ratio, applied the working solution to the PVDF membrane, and, after a few minutes, when the fluorescent bands were visible, blotted the excess substrate solution with filter paper. Then, exposed and photographed the membrane using the Tanon fully automated chemiluminescence analyzer.

### 2.7. Data Processing and Statistical Analysis

Statistical analysis was performed using SPSS version 18.0 (SPSS Inc., Chicago, IL, USA) and GraphPad Prism 8.0 (GraphPad Software Inc., La Jolla, CA, USA). Count data were analyzed using the chi-square test or Fisher’s exact test. Measurement data that followed a normal distribution were analyzed using the *t*-test, while non-normally distributed data were analyzed using the nonparametric rank-sum test. The *t*-test or nonparametric rank-sum test was also used to analyze the differences in RNA content and protein concentration between the LGBLEL and MALT groups. *p* values < 0.05 were considered statistically significant.

## 3. Results

### 3.1. Transcriptome Sequencing Results of Lymphoma and CH

**Figure 2:**
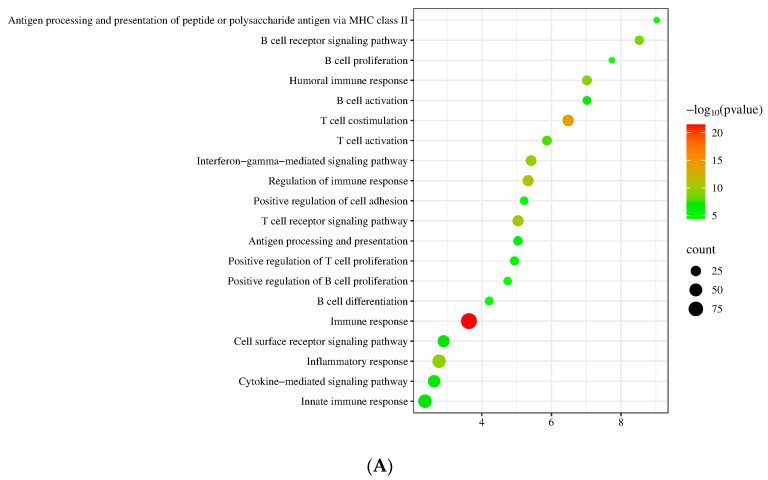

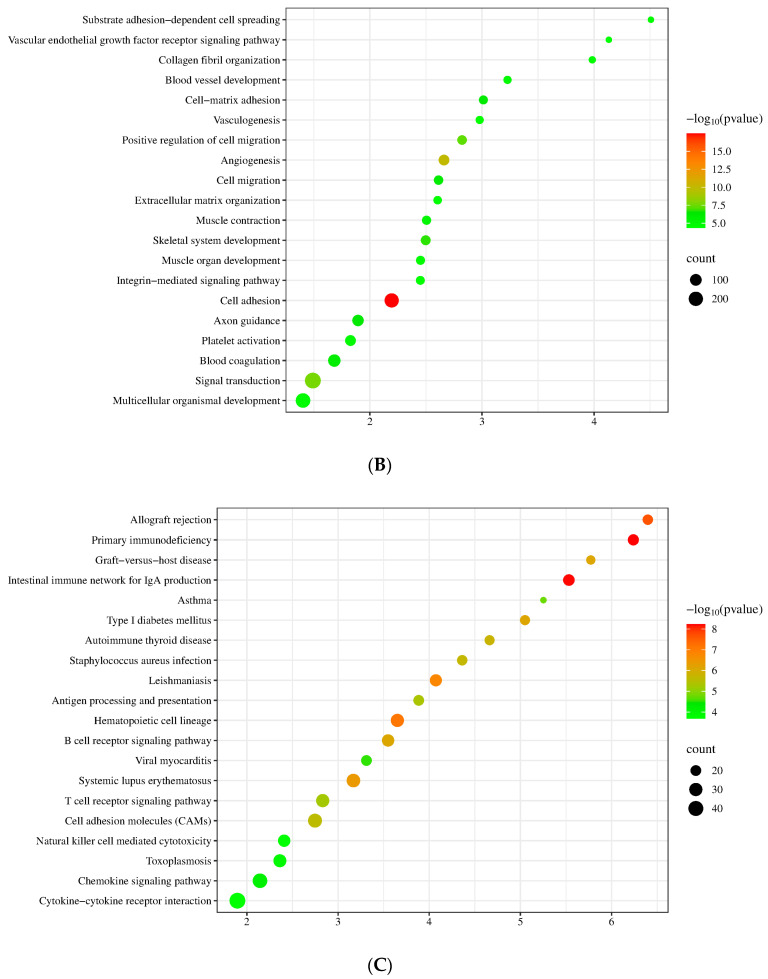

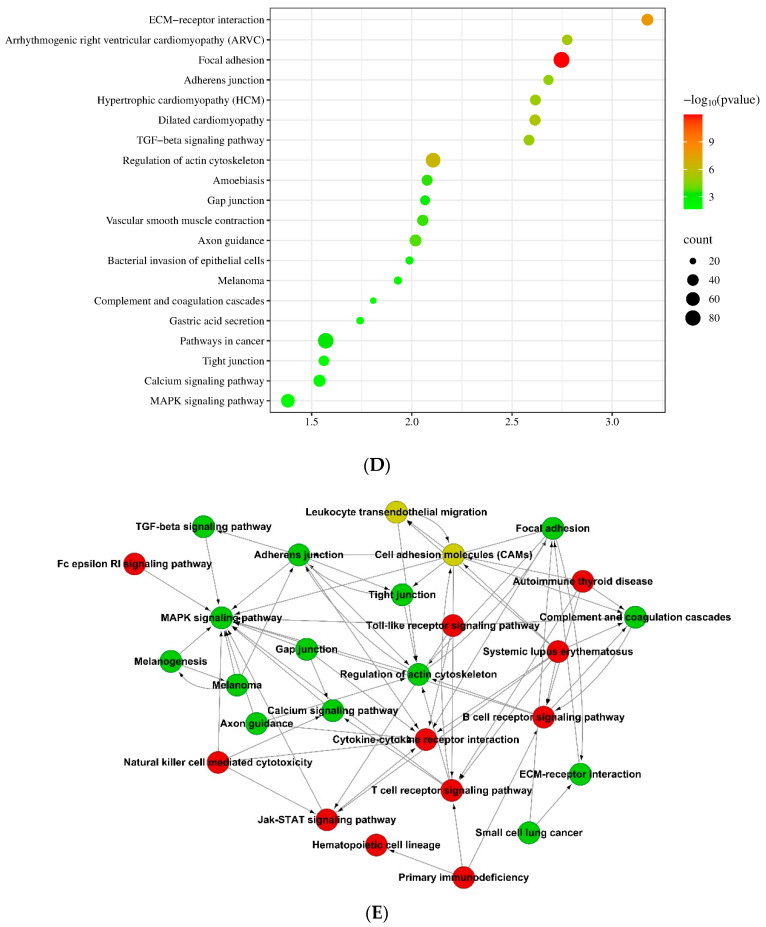
Transcriptome sequencing of lacrimal lymphoma and CH. (**A**) Top 20 enriched biological processes for upregulated DEGs. (**B**) Top 20 enriched biological processes for downregulated DEGs. (**C**) Top 20 pathway enrichment analyses for upregulated DEGs. (**D**) Top 20 pathway enrichment analyses for downregulated DEGs. (**E**) Diagram of the interaction network of differentially expressed signaling pathways (red: upregulated, green: downregulated, yellow: upregulated and downregulated).

We conducted transcriptome sequencing analysis of lymphoma and CH and identified 5409 DEGs (Supplementary S1). In comparison to the CH group, GO analysis revealed that the top 20 upregulated terms were enriched in immune response, T- and B-cell development, activation, aggregation, inflammation, and other processes, while the downregulated terms were enriched in the processes of cell adhesion, signal transduction, angiogenesis, and tissue and organ development (Figure 2A,B) (Supplementary S2). KEGG analysis results showed that the top 20 upregulated signaling pathways included BCR signaling, TCR signaling, and chemokine signaling (Figure 2C), whereas the downregulated signaling pathways included TGFβ signaling pathways, pathways in cancer, and calcium signaling pathways (Figure 2D) (Supplementary S3). The interaction network of differentially expressed signaling pathways is shown in Figure 2E. These results suggest that the chemokine signaling pathway is differentially expressed in lymphoma and CH. We analyzed the chemokine signaling pathway of lymphoma and CH and identified 38 DEGs, including *GNG4*, *CXCL14*, *CXCL9*, *GNG7*, *RASGRP2*, *CCR2*, *CCL11*, *ITK*, *GNG2*, *CCR6*, *CCL5*, *VAV2*, *PRKCD*, *CCL21*, *CCL17*, *CCL19*, *LYN*, *CCL22*, *GNB5*, *PRKCB*, *ADRBK2*, *CXCR3*, *CCR5*, *HCK*, *CSK*, *GNGT2*, *CXCR4*, *STAT1*, *CCR7*, *GRK6*, *ADCY7*, *CXCR5*, *PREX1*, *PIK3CD*, *RAC2*, *DOCK2*, *JAK3*, and *VAV1* (Supplementary S3).

### 3.2. Transcriptome Sequencing Results of Lymphoma and LGBLEL

**Figure 3:**
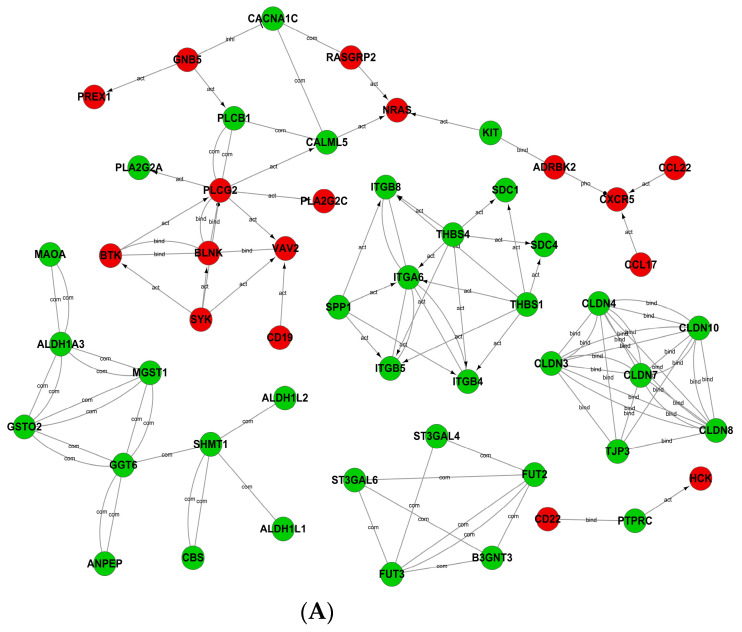

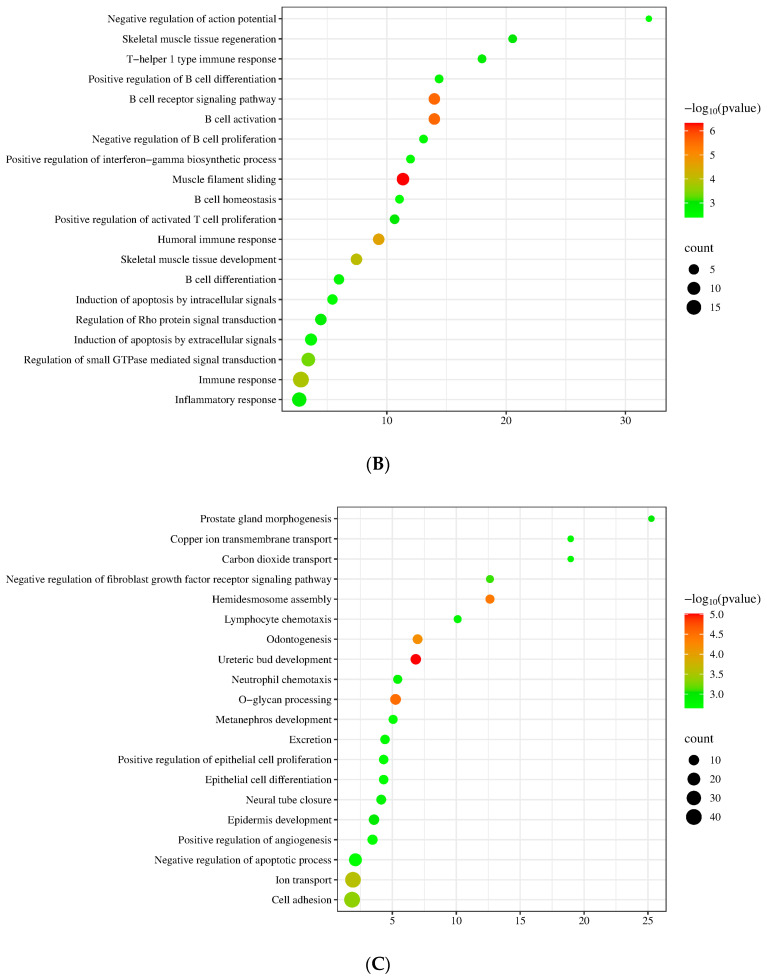

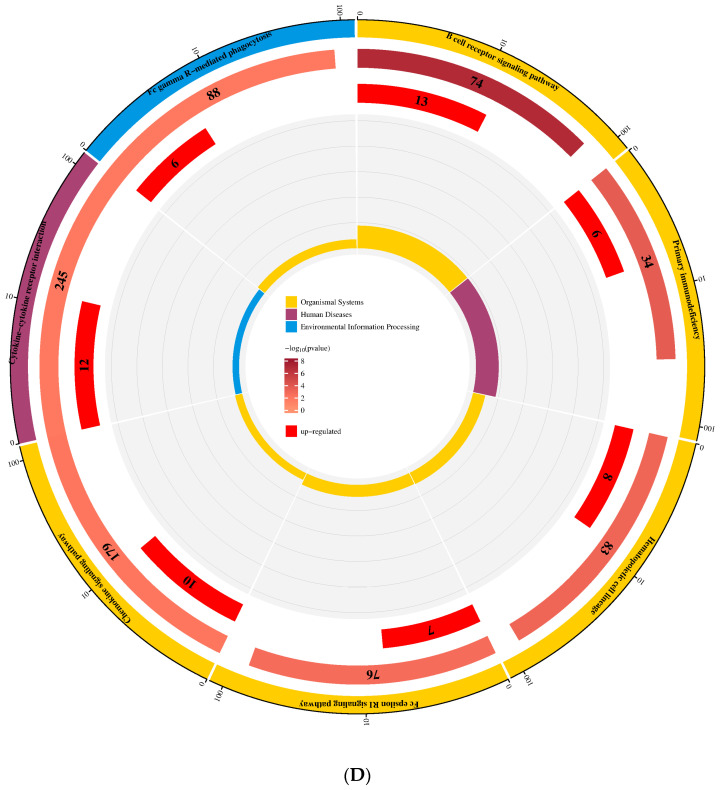

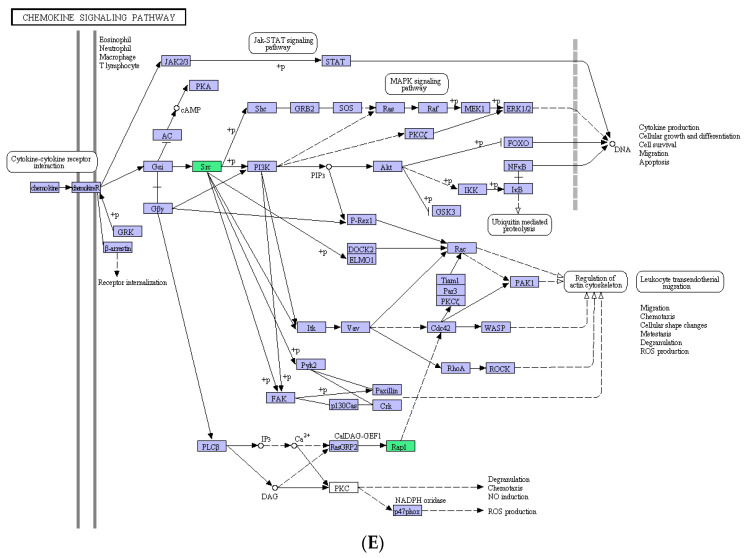
Transcriptome sequencing of lacrimal lymphoma and LGBLEL. (**A**) Interaction network diagram of DEGs (red: upregulated, green: downregulated). (**B**) Top 20 enriched biological processes for upregulated DEGs. (**C**) Top 20 enriched biological processes for downregulated DEGs. (**D**) Diagram of enrichment results of pathway with differential expression. (**E**) DEGs mapped with KEGG chemokine signaling pathway (green: downregulated; purple: upregulated).

Compared to the lymphoma group, 1301 DEGs were found in the LGBLEL group, including 439 upregulated and 862 downregulated genes (Supplementary S4). The interaction network of differential genes is shown in Figure 3A (Supplementary S5). GO analysis revealed that the top 20 upregulation terms were enriched in the BCR signaling pathway, B- and T-cell activation, development, immune response, inflammatory response, and other processes. The downregulated terms were enriched in cell development, metabolism, adhesion, and differentiation (Figure 3B,C) (Supplementary S6). KEGG analysis showed significant differences in the BCR signaling pathway, Fc epsilon RI signaling pathway, chemokine signaling pathway, and cytokine–cytokine receptor interaction (Figure 3D). Additionally, we noted 19 downregulated pathways, primarily related to salivary secretion, immune system development, cell metabolism, cell development, adhesion, and other processes (Supplementary S7). We analyzed the chemokine signaling pathway and found 10 DEGs between lymphoma and LGBLEL, including *CCL17*, *VAV2*, *CXCR5*, *NRAS*, *HCK*, *RASGRP2*, *PREX1*, *GNB5*, *ADRBK2*, and *CCL22* (Supplementary S8). Therefore, we hypothesize that the chemokine signaling pathway is upregulated in the LGBLEL group compared with the lymphoma group and may play a crucial role in its malignant transformation. A map of the DEGs with the KEGG pathways for the chemokine signaling pathway is exhibited in Figure 3E. The activation of the chemokine signaling pathway involves a diverse array of chemokines and receptors. In preliminary IHC staining experiments, we identified key proteins associated with the activation of the chemokine signaling pathway based on transcriptome sequencing results and relevant literature (Supplementary S4 and S8). These proteins included CCL28, CCR1, CXCL17, HCK, GNB5, NRAS, and VAV2.

### 3.3. Key Genes of the Chemokine Signaling Pathway Are Differentially Expressed in LGBLEL and Lymphoma

**Figure 4:**
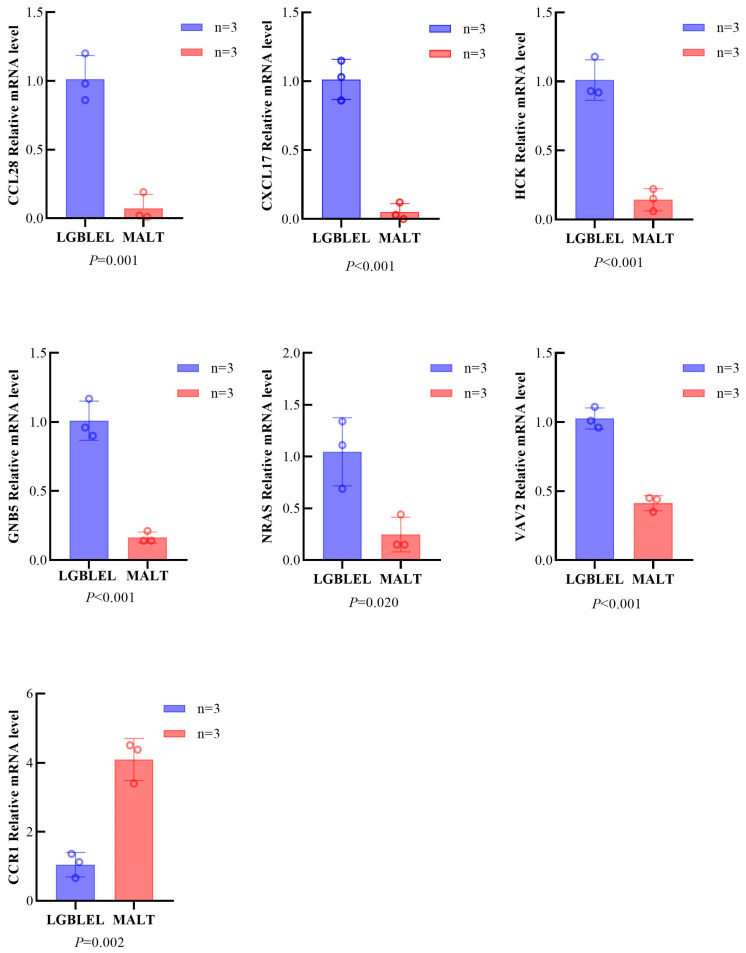
The mRNA expression levels of important genes (*CCL28*, *CCR1*, *CXCL17*, *HCK*, *GNB5*, *NRAS* and *VAV2*) in the chemokine signaling pathway. (Error: mean ± SD; test method: Wilcoxon rank-sum test).

Based on the transcriptomic sequencing results, we selected some DEGs of the chemokine signaling pathway for RT-qPCR detection. The results showed significant differences in the mRNA expression levels of *CCL28*, *CCR1*, *CXCL17*, *HCK*, *GNB5*, *NRAS*, and *VAV2*. Compared to the lymphoma group, the LGBLEL group exhibited higher expression levels of *CCL28*, *CXCL17*, *HCK*, *GNB5*, *NRAS*, and *VAV2* (*p* = 0.001, <0.001, <0.001, <0.001, 0.020, and <0.001, respectively) and lower expression of *CCR1* (*p* = 0.002) (Figure 4).

### 3.4. IHC Revealed Differential Expression of Key Proteins in the Chemokine Signaling Pathway in LGBLEL and Lymphoma

**Figure 5:**
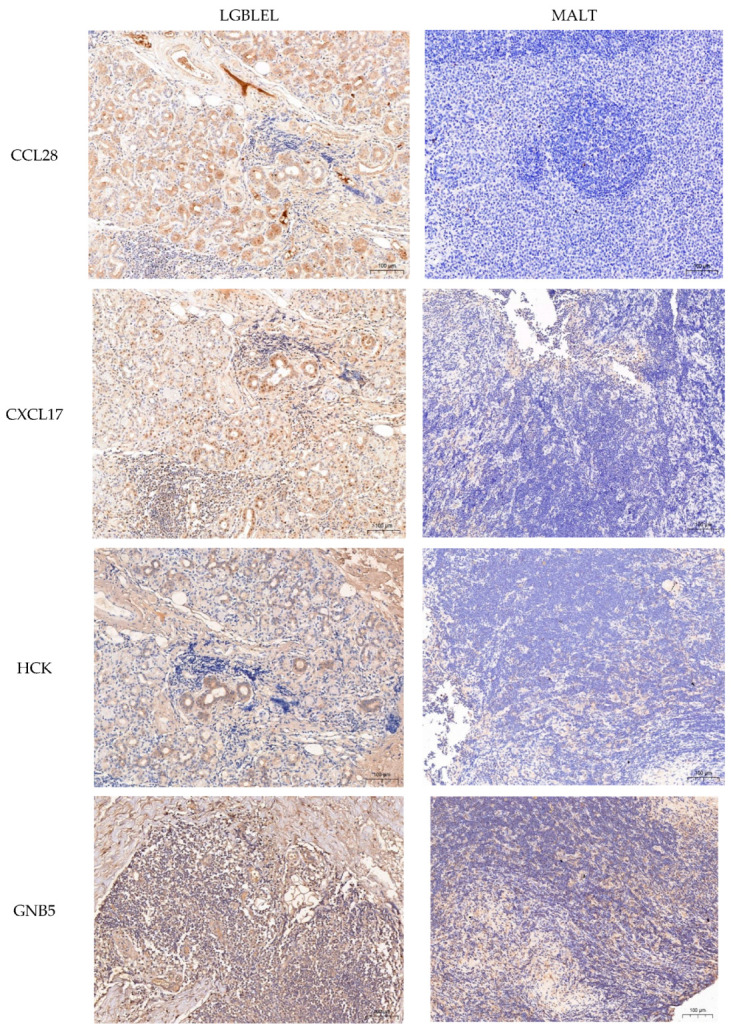

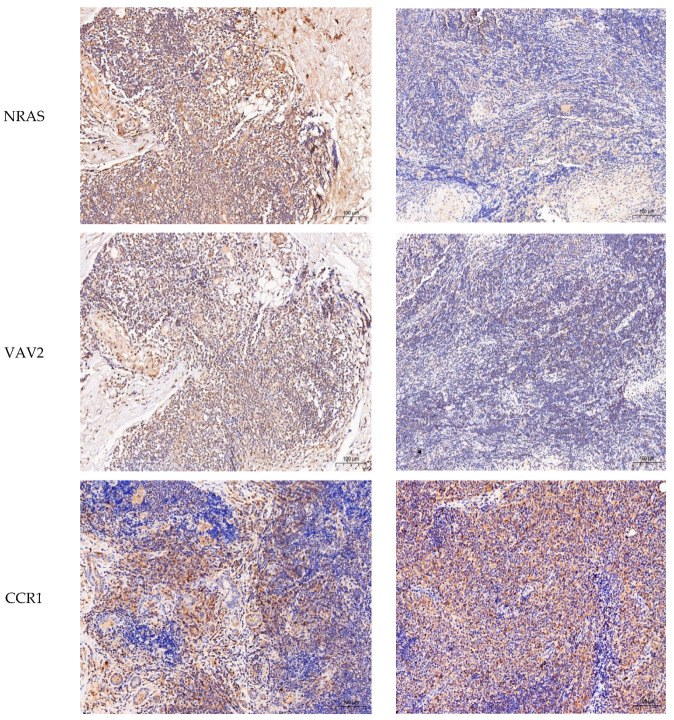
Immunohistochemical staining of proteins related to the chemokine signaling pathway in LGBLEL and MALT groups (SP method, magnification ×200). CCL28, CXCL17, HCK, GNB5, NRAS, and VAV2 were low or not expressed in the B lymphocyte infiltration region of the MALT lymphoma group, and high expression in the acinar region or lymphocyte infiltration region of LGBLEL. The expression level of CCR1 in the MALT lymphoma group was higher than that of LGBLEL.

**Figure 6:**
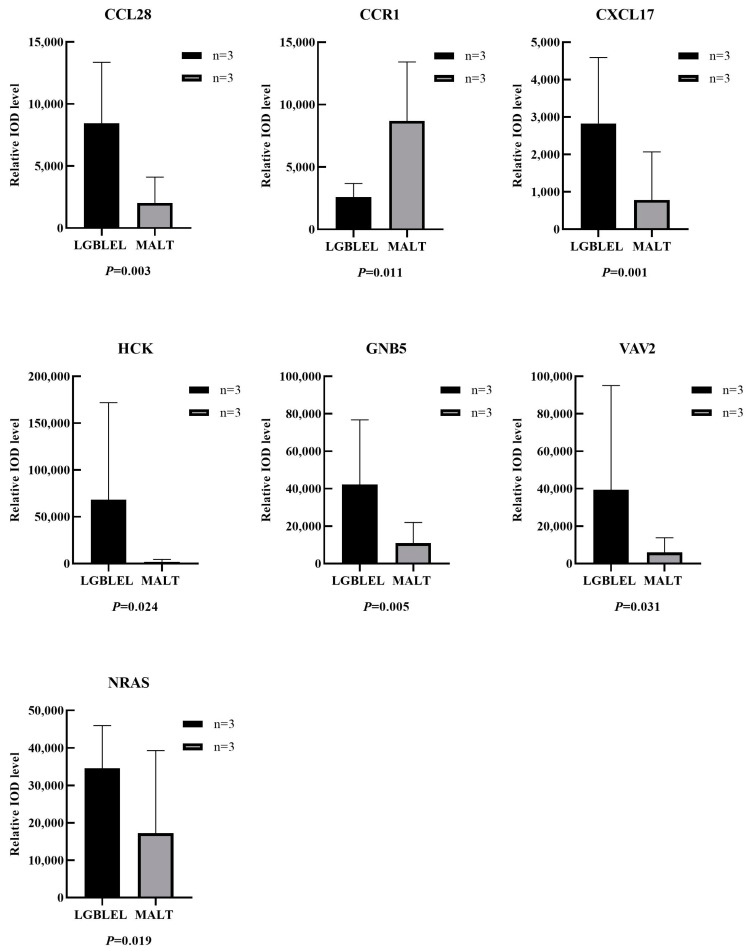
Quantitative analysis of immunohistochemical indicators. The expression level of proteins CCL28, CXCL17, HCK, GNB5, NRAS, and VAV2 in the LGBLEL was higher than that of MALT lymphoma group. The expression level of CCR1 in the MALT lymphoma group was higher than that of LGBLEL. (Error: mean ± SD; test method: Wilcoxon rank-sum test).

IHC staining showed that all indicators were highly expressed in LGBLEL acinar cells; however, in the B lymphocyte infiltrated area, with the exception of HCK, which was negatively expressed, CCL28, CCR1, CXCL17, GNB5, NRAS, and VAV2 were all positively expressed. In lymphoma groups, besides the strongly positive expression of CCR1, other indicators were either not expressed or expressed at low levels (Figure 5). Compared to the lymphoma group, the protein quantitative analysis of the LGBLEL group exhibited significantly differentially expressed CCL28, CCR1, CXCL17, HCK, GNB5, NRAS, and VAV2 (*p* = 0.003, 0.011, 0.001, 0.024, 0.005, 0.019, and 0.031, respectively) (Figure 6).

### 3.5. Western Blotting Reveals Differential Expression in the Chemokine Signaling Pathway Key Proteins in LGBLEL and Lymphoma

**Figure 7:**
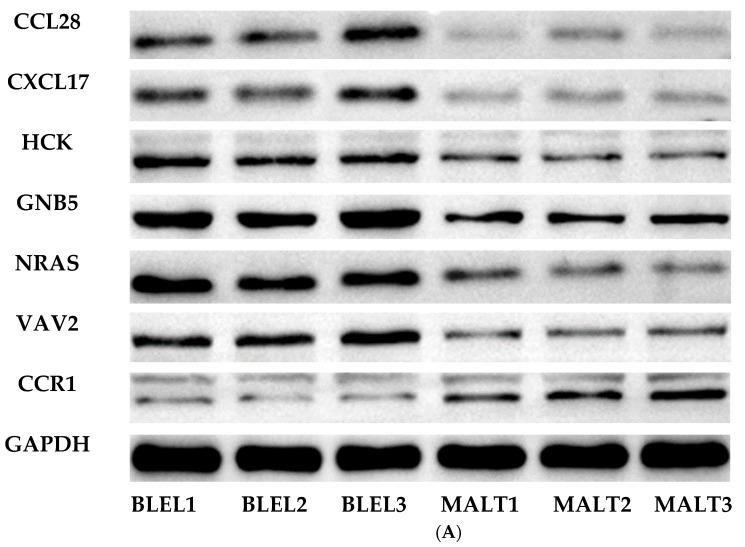

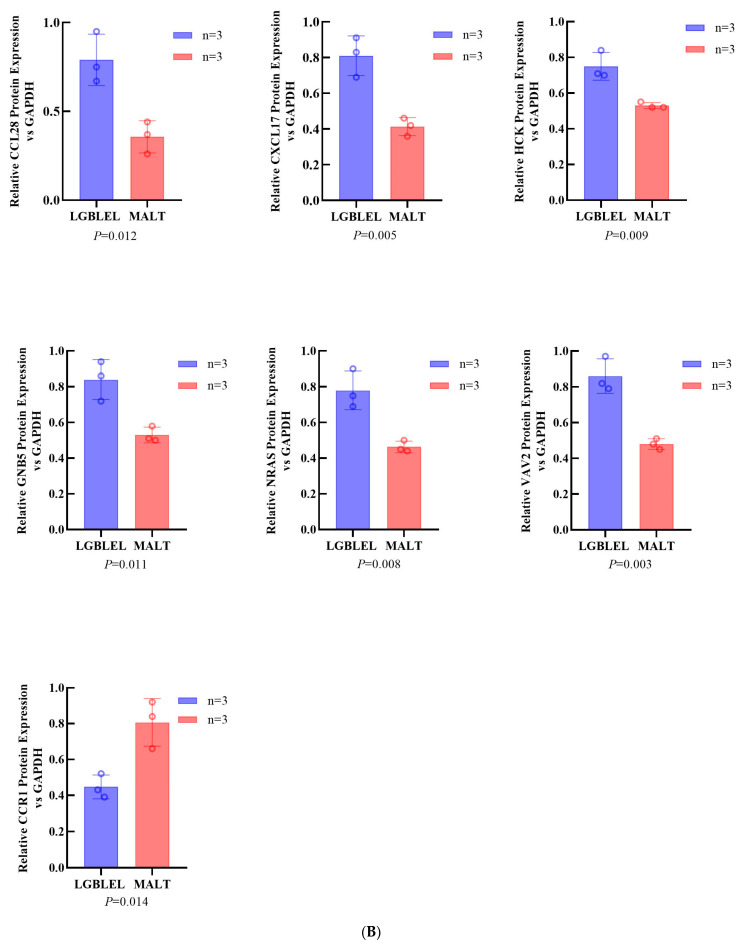
Western blotting analysis of key proteins of the chemokine signaling pathway. (**A**) Immunoblotting images of key proteins in the chemokine signaling pathway. (B) Quantitative analysis of key proteins in the chemokine signaling pathway. (Error: mean ± SD; test method: Wilcoxon rank-sum test).

WB analysis showed that CCL28, CCR1, CXCL17, HCK, GNB5, NRAS, and VAV2 were differentially expressed. Compared to the lymphoma group, the LGBLEL group showed higher expression of CCL28, CXCL17, HCK, GNB5, NRAS, and VAV2 (*p* = 0.012, 0.005, 0.009, 0.011, 0.008, and 0.003, respectively) and lower expression of CCR1 (*p* = 0.014) (Figure 7A,B).

## 4. Discussion

Chemokines are categorized into four subfamilies, including CC, CXC, CX3C, and XC. The interaction between chemokines and their G protein-coupled receptors promotes leukocyte migration and directly recruits leukocytes to the inflammatory sites, aiding in tissue repair [19]. Beyond their role in inflammation, chemokines are involved in various processes such as cell and tissue proliferation, activation, and differentiation; extracellular matrix remodeling; angiogenesis; and tumor metastasis [20]. They are also critical in the pathogenesis of IgG4-RD. The literature indicates that SDF-1/CXCL12 can regulate B-cell development, neovascularization, and fibrosis and may play a role in IgG4-RD [21]. Additionally, the CCL18-CCR8 axis may contribute to the pathogenesis of IgG4-RD by promoting cell chemotaxis, inducing fibrosis, and stimulating IgG4 production. CCL18 also serves as a valuable biomarker for assessing both disease activity in IgG4-RD and patient response to treatment [22,23]. Targeting CXCR5 and its ligand, CXCL13, presents a potential therapeutic strategy for patients with refractory IgG4-RD [24]. LGBLEL, a form of IgG4-ROD, has not previously been linked to the chemokine signaling pathway.

Chemokines and their receptors also play a crucial role in the pathogenesis of various lymphomas, such as diffuse large B-cell lymphoma, follicular lymphoma, and T-cell lymphoma [25,26]. Research has demonstrated that chemokines are important for distinguishing lymphoma from other pathologically similar conditions. Commonly recognized markers include CCL17, CXCR4, CXCL9, CXCL10, CXCR3, CXCL13, and CCL11 [25,26,27,28]. In studies on primary Sjogren’s syndrome, CXCL13 and CCL11 have been linked to chronic B-cell activation, disease activity, and the development of lymphoma [29,30]. Additionally, CXCL13 and CCL21 directly contribute to reactive lymphocyte proliferation, while CXCL12 is associated with epithelial infiltration and malignant B-cell components, potentially regulating the survival of malignant B cells [31]. Furthermore, CXCR3, CXCR4, and CXCR5 have been implicated in the pathogenesis of MALT lymphoma [32,33].

We employed IHC and WB for protein quantification, with WB offering greater accuracy for detecting protein levels, while IHC allowed for the localization of protein expression. This localization helped us better evaluate protein changes during the transformation of B lymphocytes. Our findings showed that CCL28 was highly expressed in the acinar and the lymphocyte infiltration regions of LGBLEL but absent in the lymphoma group. Similarly, CXCL17 was highly expressed in these regions in LGBLEL but not in the lymphoma group. CCR1 showed low positively in the acinar and lymphocyte infiltration area of LGBLEL but was highly positively expressed in lymphoma. CCL28 has been shown to selectively attract lymphocyte subsets and are involved in the mucosal homing of B and T cells [34]. It has also been reported to attract eosinophils and promote plasma cell infiltration and facilitate monocyte migration in classic Hodgkin’s lymphoma tissue or inflammatory lesions, contributing to inflammation or tumorigenesis [35,36]. CXCL17, an orphan chemokine, acts as a strong chemoattractant for monocytes, dendritic cells, and macrophages [37]. It may also have anti-inflammatory activity, as previous studies have showed that increased CXCL17 expression can inhibit the release of pro-inflammatory cytokines, thus playing a role in inhibiting inflammatory responses [38]. CXCL17 is also involved in the pathogenesis of diffuse large B-cell lymphoma, where it serves as a prognostic marker [39]. CCR1, a member of the C-C motif chemokine receptor subfamily, is expressed in monocytes, natural killer cells, and immature bone marrow cells. Studies have indicated that the expression level of CCR1 in MALT lymphomas is higher than that in inflammatory lesions, as well as being higher in high-grade lymphomas such as diffuse large B-cell lymphomas, which seems to be related to the high-grade transformation of lymphomas [40,41]. Based on these findings, we speculated that the loss of CCL28 and CXCL17 expression and the increase in CCR1 expression in LGBLEL tissue may be related to malignant transformation and could serve as an effective prognostic indicator.

HCK, GNB5, NRAS, and VAV2 are key genes in the chemokine signaling pathway, but there have been no reports on the role of this pathway in the malignant transformation mechanism of LGBLEL. Lymphocytes play a crucial role in the pathogenesis of both LGBLEL and lymphoma. However, lymphocyte trafficking and homing are tightly controlled by signaling pathways and are mediated through cytokines, chemokines, their respective receptors, and adhesion molecules [42]. Therefore, we speculate that, in LGBLEL, elevated levels of certain chemokines such as CCL28 contribute to pro-inflammatory effects by activating the chemokine signaling pathway and recruiting a large number of infiltrating lymphocytes. Under the regulation of autoimmune mechanisms, the increased expression of CXCL17 plays a role in suppressing inflammatory responses. Prolonged chronic inflammation may lead to clonal expansion of lymphocytes, resulting in lymphoma. In these abnormally activated B lymphocytes, the expression of chemokines and their receptors is relatively deficient or elevated, leading us to believe that these factors may be related to the malignant transformation of LGBLEL.

The mechanism underlying the malignant transformation of LGBLEL is highly complex and involves multiple signal pathways. In this study, key genes from the chemokine signaling pathway—*HCK*, *GNB5*, *NRAS*, and *VAV2*—were validated, confirming their differential expression between LGBLEL and lymphoma and suggesting their important role in the malignant transformation of LGBLEL. Moreover, we identified CCL28, CXCL17, and CCR1 as potential markers for monitoring malignant transformation and aiding in differential diagnosis. Since MALT lymphoma is the most common pathological type of malignant transformation in LGBLEL, patients with MALT lymphoma were selected as the experimental group during the verification stage. Our findings related to the transformation into high-grade lymphoma will be further verified in future studies, providing a foundation for understanding the malignant transformation mechanisms of LGBLEL.

## Figures and Tables

**Table 1:** Characteristics of patients with LGBLEL, lymphoma, and CH for transcriptome sequencing.

Group	Mean Age (Years Old)	Sex (Male: Female)
LGBLEL (n = 15)	42.60 ± 8.48 (range, 27–57)	1:6.5
Lymphoma (n = 14)	57.43 ± 11.06 (range, 38–78)	2.5:1
CH (n = 9)	48.67 ± 7.09 (range, 37–55)	1:3.5

**Table 2:** The statistical differences in age and gender among LGBLEL, lymphoma and CH.

*p* Value	LGBLEL vs. Lymphoma	Lymphoma vs. CH	LGBLEL vs. CH
Mean age (years old)	0.000 ^#^	0.016 ^#^	0.069 ^#^
Sex (male: female)	0.003 *	0.036 *	0.615 *

Note: “*” stands for Fisher’s exact probability method; “^#^” stands for Wilcoxon rank-sum test.

**Table 3:** Sequences of real-time PCR primers.

Gene	Primer Sequence 5′-3′
*h GAPDH_F*	GCCTTCCGTGTCCCCACTGC
*h GAPDH_R*	GGCTGGTGGTCCAGGGGTCT
*h CCL28_F*	GCCCTACATGCCTCAGAAG
*h CCL28_R*	CTTAACAGTATGGTTGTGCGG
*h CXCL17_F*	CTGTTGCTGCCACTAATGC
*h CXCL17_R*	GCTCTCAGGAACCAATCTTTG
*h HCK_F*	GGAGCCCATCTACATCATCA
*h HCK_R*	CCGTGTACTCGTTGTCCTCA
*h NRAS_F*	CAATCCAGCTAATCCAGAACC
*h NRAS_R*	TGTTTCCCACTAGCACCATAG
*h GNB5_F*	GTTCTGTGTACCCCTTGACG
*h GNB5_R*	GGCTTTCTTGTCACATCCC
*h VAV2_F*	ACGAGGACATCATCAAGGTG
*h VAV2_R*	GGTAGTACTTGGCCTCGGTC
*h CCR1_F*	GGAATTCACTCACCACACCTG
*h CCR1_R*	ACGGACAGCTTTGGATTTCT

## Data Availability

All raw data is stored in https://www.jianguoyun.com/p/DR_JGaQQxe7yCxigxaoFIAA (accessed on 4 March 2024).

## Supplementary Materials

The following supporting information can be downloaded at: https://www.mdpi.com/article/10.3390/cimb46100652/s1.

## Abbreviations

LGBLEL	lacrimal gland benign lymphoepithelial lesion
CH	cavernous hemangioma
RT-qPCR	Real-time quantitative polymerase chain reaction
IHC	immunohistochemical
WB	Western blotting
IgG4-ROD	immunoglobulin G4-related ophthalmic disease
IgG4-RD	IgG4-related disease
MALT	muco-associated lymphoid tissue
DEGs	differentially expressed genes
FDR	false discovery rate
GO	Gene Ontology
KEGG	Kyoto Encyclopedia of Genes and Genomes
